# Metabolic Health, Insulin, and Breast Cancer: Why Oncologists Should Care About Insulin

**DOI:** 10.3389/fendo.2020.00058

**Published:** 2020-02-20

**Authors:** Lisa D. Yee, Joanne E. Mortimer, Rama Natarajan, Eric C. Dietze, Victoria L. Seewaldt

**Affiliations:** City of Hope Comprehensive Cancer Center, Duarte, CA, United States

**Keywords:** breast cancer, insulin, metabolic health, metformin, TNBC (Triple negative breast cancer)

## Abstract

Studies investigating the potential link between adult pre-menopausal obesity [as measured by body mass index (BMI)] and triple-negative breast cancer have been inconsistent. Recent studies show that BMI is not an exact measure of metabolic health; individuals can be obese (BMI > 30 kg/m^2^) and metabolically healthy or lean (BMI < 25 kg/m^2^) and metabolically unhealthy. Consequently, there is a need to better understand the molecular signaling pathways that might be activated in individuals that are metabolically unhealthy and how these signaling pathways may drive biologically aggressive breast cancer. One key driver of both type-2 diabetes and cancer is insulin. Insulin is a potent hormone that activates many pathways that drive aggressive breast cancer biology. Here, we review (1) the controversial relationship between obesity and breast cancer, (2) the impact of insulin on organs, subcellular components, and cancer processes, (3) the potential link between insulin-signaling and cancer, and (4) consider time points during breast cancer prevention and treatment where insulin-signaling could be better controlled, with the ultimate goal of improving overall health, optimizing breast cancer prevention, and improving breast cancer survival.

Over the past 20 years, much of the world, has experienced a growing obesity epidemic. Once a disease of the wealthy, obesity now disproportionately effects women and the poor. Possible contributors include disparities in income that promote consumption of inexpensive high calorie foods with low nutritional value (1), lack of access to healthy food sources (food deserts) (2) (https://www.nap.edu/catalog/12623/the-public-health-effects-of-food-deserts-workshop-summary), and adoption of a sedentary lifestyle. Along with the rise in obesity, there has been a rise in gestational diabetes and type-2 diabetes. Much research is focusing on the potential link between obesity, type-2 diabetes, and breast cancer, as increasingly women who are at risk for type-2 diabetes are also at risk for breast cancer. There has been much work investigating the potential link between obesity [as measured by body mass index (BMI)] and breast cancer. Results have been conflicting, likely reflecting the complex relationship between BMI and metabolic health. Recent studies show that BMI is not an exact measure of metabolic health. Individuals can be obese (BMI > 35 kg/m^2^) and metabolically healthy or lean (BMI < 25 kg/m^2^) and metabolically unhealthy. Consequently, there have been efforts to better understand the molecular signaling pathways that might be activated in individuals that are metabolically unhealthy. One key driver of both type-2 diabetes and cancer is insulin. Insulin is a potent hormone that activates many pathways that drive aggressive breast cancer biology. Here, we aim to review (1) the controversial relationship between obesity and breast cancer, (2) the impact of insulin on organs, subcellular components, and cancer processes, (3) the potential link between insulin-signaling and cancer, and (4) consider time points during breast cancer prevention and treatment where insulin-signaling could be better controlled, with the ultimate goal of improving overall health, optimizing breast cancer prevention, and improving breast cancer survival.

## Complex Relationship Between Obesity and Breast Cancer

### Obesity and Breast Cancer

Increased adiposity in childhood has been consistently associated with a decreased risk of pre- and post-menopausal breast cancer. Conversely, increased adiposity after menopause is associated with increase of risk (3–26). However, the majority of these have been case-control studies and have not assessed breast cancer-subtype. Meta-analyses of aggregated studies were (1) not uniform in their age at BMI measurement, attained age of participants, and degree of adjustment for potential confounding factors, and (2) not stratified by other risk factors (4–7, 17–20, 27). A summary of trials testing for the association between obesity and breast cancer (and findings) is presented in Table 1A.

**Table 1:** The association between: **(A)** Obesity and ER+ or ER- Breast Cancer and **(B)** Metabolic Parameters and Cancer.

**(A)**			
**Study**	**References**	**Relative risk of breast cancer, obese vs. normal weight**	**Comments**
Premenopausal breast cancer collaborative	(27)	Age 18–24 RR = 0.77/5 kg/m^2^ increase in BMI (95% CI, 0.73–0.80) Age 45–54 RR+0.88/5 kg/m^2^ increase in BMI (95% CI, 0.86–0.91)	
Carolina breast cancer study	(28)	Premenopausal RR = 1.80 (95% CI, 1.0–3.4) Post-menopausal RR = 2.70 (95% CI, 1.3–5.4)	For high WHR and basal type TNBC
Appalachian study	(29)	Significant association between obesity and incidence	For TNBC
Women's CARE study	(30)	Inverse association BMI at age 18 and premenopausal BC Positive association current BMI and post-menopausal BC	For ER-/PR- BC For ER+/PR+ BC
Black women's health study	(31)	Inverse association BMI at age 18 and pre- or post-menopausal BCInverse association current BMI and premenopausal BC	
Women's circle of health study	(32, 33)	Inverse association BMI and post-menopausal BC Association with premenopausal BC	For ER-/PR- BC For high WHR
AMBER consortium	(34)	Post-menopausal RR = 1.31 (95% CI, 1.02–1.67) Post-menopausal RR = 0.60 (95% CI, 0.39–0.90) Inverse association premenopausal BMI with pre- or post-menopausal BC Association with premenopausal BC	For ER+ BC For TNBC For ER+ BC-all BC For high WHR and ER+ BC
**(B)**			
**Study**	**References**	**Metabolic parameters and cancer**	**Comments**
ADA/ACS Consensus Report	(35)	Positive association between diabetes and cancer	For all cancer
Barone et al.	(36)	RR = 1.41 (95% CI, 1.28–1.55)	For all cancer
Danker et al.	(37)	RR = 1.37 (95% CI, 0.94–2.00)	Mortality for all cancer
Hemkens et al.	(38)	Positive association between insulin dose and risk of cancer	For all cancer
Kabat et al.	(39)	Positive association between poor metabolic health and BC risk	For obese women and all post-menopausal BC
Sister Study	(40)	Positive association between poor metabolic health and BC risk	For normal weight women and all post-menopausal BC
Iyengar et al.	(41)	Positive association between high body fat, poor metabolic health, and risk of invasive BC	For normal weight women and all post-menopausal BC
Women's Health Initiative	(42)	Positive association between hyperinsulinemia and BC risk	For post-menopausal BC

The Premenopausal Breast Cancer Collaborative group recently published a multicenter analysis used pooled individual-level data from 758,592 premenopausal women from 19 prospective cohorts to estimate hazard ratios (HRs) of premenopausal breast cancer in association with BMI from ages 18 through 54 years (27). Results of this study provide evidence increased adiposity was associated with a reduced risk of premenopausal breast cancer at a greater magnitude than previously shown and across the entire distribution of BMI. The strongest associations of risk were observed for BMI in early adulthood. Among the 758,592 premenopausal women (median age, 40.6) included in the analysis, inverse linear associations of BMI with breast cancer risk were higher for BMI age 18–24 years [HR per 5 kg/m^2^ (5.0-U) difference, 0.77; 95%CI, 0.73–0.80] than for BMI age 45–54 years (HR per 5.0-U difference, 0.88; 95%CI, 0.86–0.91). The investigators observed a 4.2-fold risk gradient between the highest and lowest BMI categories (BMI ≥35.0 vs. <17.0) at ages 18–24 years. Associations between BMI and breast cancer were stronger for estrogen receptor–positive (ER+) and/or progesterone receptor–positive (PR+) than for hormone receptor–negative (ER–) breast cancers.

### Obesity and Triple Negative Breast Cancer (TNBC)

The potential association between obesity and TNBC has been a subject of intense research study. As outlined below, studies investigating the potential relationship between obesity and TNBC have been inconsistent. This is a summary of some of the key studies that have been recently published.

The Carolina Breast Cancer Study is a North Carolina population-based case-controlled study of breast cancer, conducted in three phases (28). The current study phase, Phase 3 (years 2008–2014), includes women resident in 44 North Carolina Counties (28), employing randomized recruitment to oversample African-American/Black women and women under age 50 (28). Waist to hip ratio (WHR) was compared between the highest (≥0.84) and lowest (<0.77) groups vs. the basal-type subset of TNBC. There was an increased risk [odds ratio (OR) = 2.3; 95% CI, 1.4–3.6] for basal-type TNBC in women with higher WHR (43). Premenopausal women (OR = 1.8; 95% CI, 1.0–3.4) and post-menopausal women (OR = 2.7; 95% CI, 1.3–5.4) with the highest WHR had increased risk of developing basal-type TNBC compared to the lowest WHR (43). Basal-type breast cancer was observed to be highest among premenopausal African-American/Black women (43). There was no significant association between increased BMI (defined as BMI ≥ 25 kg/m^2^) and basal-type TNBC.

The Appalachian Study investigated the potential association between obesity (defined as BMI ≥ 30 kg/m^2^) and TNBC in 620 predominantly non-Hispanic White women in rural Appalachia. This study reported a significant association between obesity and the incidence of TNBC (29).

Women's CARE study is a case-controlled study of BMI and breast cancer risk in non-Hispanic White women and African-American/Black women. The Women's CARE study reported (1) an inverse association between a woman's BMI at age 18 and premenopausal ER–/PR– breast cancer and (2) a positive association between current BMI and post-menopausal ER+/PR+ breast cancer (30).

The Black Women's Health Study (BWHS) is a prospective study among African-American/Black women across the United States (31). The study was established in 1995, with 59,000 African-American/Black women responding to a 14-page health questionnaire. The BWHS tested for the potential association between body size and breast cancer. In the BWHS, high BMI at age 18 was associated with reduced risk of both pre- and post-menopausal breast cancer, and current BMI was inversely associated with premenopausal cancer (13). There was also a trend toward a positive association between high BMI and ER+/PR+ breast cancer.

The Women's Circle of Health Study is a multi-site case–control study in New York City and New Jersey that aims to identify risk factors for early aggressive breast cancers in African-American/Black and non-Hispanic European-American women (32, 33). Recently, the Women's Circle of Health Study observed significant inverse associations of high BMI with ER–/PR– breast cancer among post-menopausal women. Similar to the Carolina Breast Study, increased WHR was associated with an increased risk of premenopausal breast cancer after adjustment for BMI (32, 33, 43).

The Premenopausal Breast Cancer Collaborative group (as described above) observed an inverse association between premenopausal obesity and ER+/PR+ breast cancer (27). In contrast, BMI at ages 25–54 years was not consistently associated with TNBC or ER- breast cancer (27).

The African-American Breast Cancer Epidemiology and Risk (AMBER) Consortium was formed, in part, to investigate the inconsistent and confusing results generated in individual studies testing for the potential association between obesity (measured by BMI and/or WHR) and TNBC. The AMBER Consortium (34) brings together four important, highly diverse cohorts: (1) Carolina Breast Cancer Study (43), (2) Women's Circle of Health Study (30), (3) Black Women's Health Study (31), and (4) Multiethnic Cohort Study (44). The AMBER Consortium found that the impact of general and central obesity varied by menopausal status and hormone receptor subtype in African-American/Black women (34). In post-menopausal women, higher recent BMI was associated with increased risk of ER+ cancer (OR: 1.31; 95% CI: 1.02–1.67 for BMI ≥ 35 vs. <25 kg/m^2^) and with a decreased risk of TNBC (OR: 0.60; 95% CI: 0.39–0.93 for BMI ≥ 35 vs. <25). Adult premenopausal women with increased BMI had a decreased incidence of (1) premenopausal ER+ breast cancer and (2) all subsequent post-menopausal breast cancer (all subtypes) (34). In adult premenopausal women, high WHR was associated with an increased risk of premenopausal ER+ breast cancer (OR: 1.35; 95% CI: 1.01–1.80) and all subsequent post-menopausal breast cancer (all subtypes) (OR: 1.26; 95% CI: 1.02–1.56 (34). The investigators concluded that in African-American/Black women, there were different mechanisms for the associations between adiposity and TNBC vs. ER+ breast cancers (34).

## Insulin Overview, Target Organs, and Activation of Cellular Signaling

### Insulin

**Figure 1:**
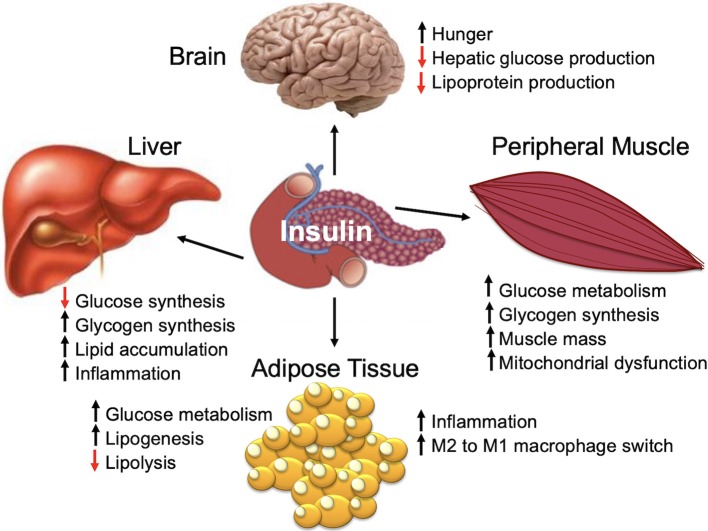
Insulin is a major regulator of metabolism and organ function.

Insulin is a peptide hormone that is produced by the pancreatic islet beta cells in response to an increase in serum glucose [for a comprehensive review, see Haeusler et al. (45)]. Insulin is a master regulator of energy storage and metabolism and has key and complex effects in the liver, muscle, brain, and fat (Figure 1) (45). Insulin stimulates glucose uptake by muscle and adipose tissue (45). In the liver, insulin inhibits gluconeogenesis and release of glucose (45). High insulin levels stimulate both the liver and muscle to store excess glucose (45). In addition to regulating the global glucose energy balance, in adipocytes, insulin promotes fatty acid transport from the blood stream, promotes storage of fat (lipogenesis), and inhibits fat breakdown (lipolysis) (45). Insulin facilitates storage of energy that can be mobilized when insulin levels are low (fasting) (45).

### Cellular Signaling

The majority of mammalian cell types express the insulin receptor and, consequently, are impacted by insulin-signaling (45). In addition to pancreatic islet beta cells, hepatocytes, and adipocytes, insulin also impacts neurons, endothelial cells, and immune cells (45).

**Figure 2:**
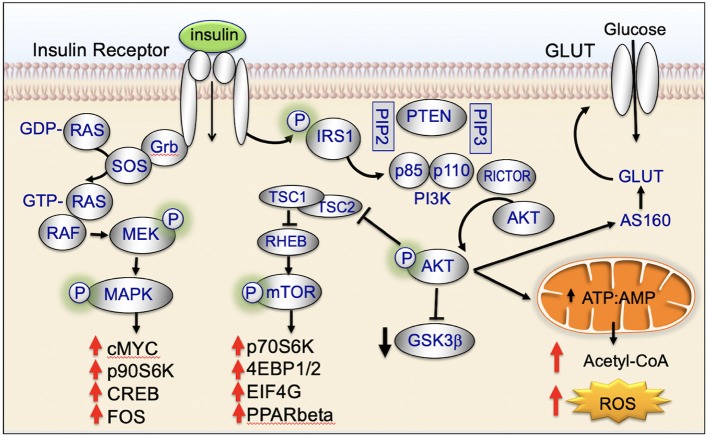
Insulin signaling, glucose uptake, and cancer processes.

Insulin binds to the insulin receptor and activates cell signaling pathways that are key regulators of cellular homeostasis. These signaling pathways are dysregulated in the majority of biologically aggressive cancers (45). Under nutrient-rich circumstances, insulin is released and binds to the insulin receptor (45). Binding of insulin promotes tyrosine phosphorylation of the insulin receptor and insulin receptor substrate (IRS) (45). IRS in turn phosphorylates phosphatidyl inositol-3-kinase (PI3K) and activates downstream AKT/mTOR-network signaling (45). Insulin also activates insulin/insulin-like growth factor-1 (IGF-1)-signaling (45). IGF-1 binds to the IGF-1 receptor (IGF-1R) leading to downstream phosphorylation cascades that activate: (1) PI3K/AKT/mTOR-network signaling and (2) RAS/RAF/mitogen activated protein kinase (MAPK) (Figure 2) (45).

## Hyperinsulinemia, Insulin-Resistance, and Gestational Diabetes

### Hyperinsulinemia

**Figure 3:**
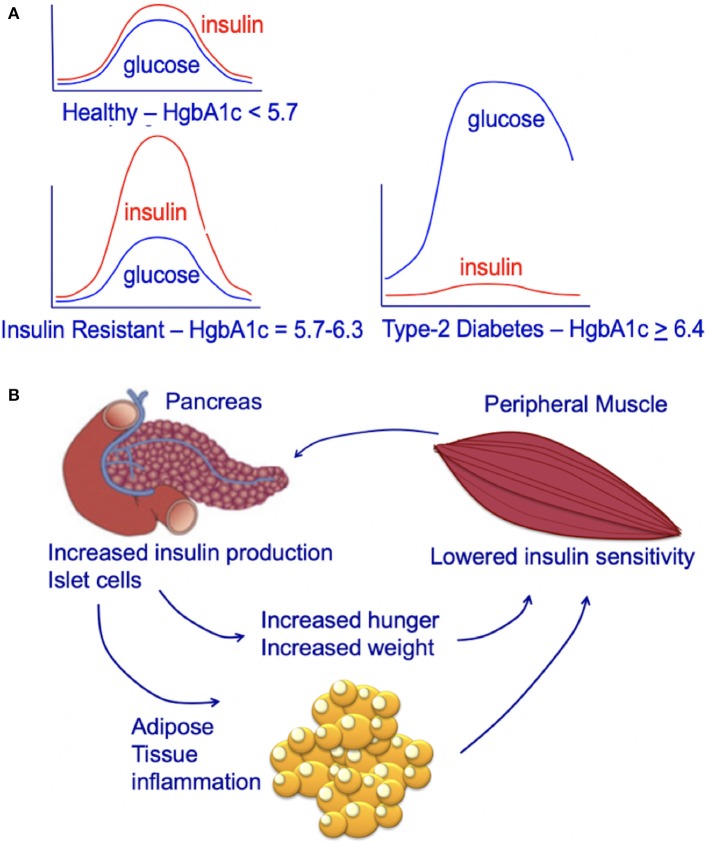
**(A)** Circulating insulin and glucose levels in healthy individuals (Healthy), insulin-resistant individuals (Resistant), and individuals with type-2 diabetes (Diabetes) at baseline and at 2 h after eating. Individuals with type-2 diabetes can experience beta-cell failure and an associated decline in serum insulin levels. This beta-cell failure/decline insulin is highly variable and time-dependent. For an excellent review see Roden and Shulman (46). **(B)** Impact of insulin-resistance on pancreatic islet cells, peripheral muscle, and individual. Insulin resistance in peripheral muscle tissue results in increased insulin demands from the pancreas. Increased circulating insulin drives hunger and increases weight, leading to a positive feedback loop that increases the chance of an individual developing type-2 diabetes.

Chronic high caloric intake (overeating) disrupts the balance between energy storage and consumption resulting in pre-diabetes (insulin resistance) and, ultimately, type-2 diabetes (45). Overeating, results in chronically high serum insulin and desensitization of skeletal muscle to insulin (45). With progressive de-sensitization (insulin-resistance), the pancreas is required to produce increasingly higher levels of insulin (hyperinsulinemia) to achieve glucose homeostasis and prevent hyperglycemia (Figure 3A) (45).

The increasing demands on the pancreatic islet beta cells that occur during hyperinsulinemia (insulin-resistance) contribute to the subsequent development of type-2 diabetes (45). Obesity and insulin-resistance initially results in high fasting insulin; this high level of insulin is thought to be due to beta-cell compensation in the face of progressive insulin-resistance, resulting in hyperinsulinemia with normal glucose tolerance (47, 48). However, after type-2 diabetes develops, some individuals develop progressive beta-cell dysfunction and failure associated with a decrease in fasting insulin (Figure 3A) (48, 49). This decrease in fasting insulin is observed in both animal models (50, 51) and humans (46). In humans, the decrease in fasting insulin has been shown to be proportional to the number of years an individual has had type-2 diabetes (52, 53). However, not all individuals with type-2 diabetes develop beta-cell dysfunction/mass and insulin production; some individuals with type-2 diabetes may have higher than normal fasting insulin (despite this increased fasting insulin not matching physiologic needs) (54). Genetics and genetic ancestry are thought to play a key role in determining whether individuals with type-2 diabetes exhibit a progressive (1) loss or (2) increase in insulin production (47, 54, 55). For a further discussion of this complex emerging area of research see the excellent new review article by Roden and Schulman (46).

### Insulin-Resistance and Gestational Diabetes

Insulin-resistance is diagnosed when an individual has impaired fasting glucose and/or impaired glucose tolerance that does not meet the threshold for diabetes (56). Insulin-resistance is diagnosed by (1) serum hemoglobin A1c (HgbA1c) of 5.7–6.3% or (2) the more complex glucose tolerance test (baseline serum glucose and insulin, oral glucose load, 2 h serum glucose and insulin (56). Measurement of serum glucose alone, is many times inadequate to detect insulin-resistance as serum glucose levels are typically only mildly elevated. The pathology of insulin-resistance lies in the high levels of insulin required to achieve these mildly elevated glucose levels. Unless treated, insulin-resistance frequently leads to type-2 diabetes (56). Type-2 diabetes occurs when the pancreas cannot keep up with demand for insulin; ultimately the pancreatic islet beta cells die, the pancreas fails (insulin-dependent type-2 diabetes), and glucose levels rise.

Gestational diabetes occurs during pregnancy when a woman is unable to make sufficient insulin for herself and her developing infant, resulting in glucose intolerance (56). Women with gestational diabetes have glucose tolerance that is impaired but does not meet the degree of impairment required for the diagnosis of diabetes (56). The diagnosis gestational diabetes is typically made by two step testing at approximately 24–28 weeks gestation (56). However, some women have insulin resistance before they become pregnant. While gestational diabetes is linked with obesity, it can occur in women who are normal weight (57, 58). Women with gestational diabetes are at increased risk for adverse pregnancy outcomes, including fetal macrosomia, pre-eclampsia/hypertensive disorders in pregnancy, and shoulder dystocia (https://extranet.who.int/rhl/topics/preconception-pregnancy-childbirth-and-postpartum-care/antenatal-care/who-recommendation-diagnosis-gestational-diabetes-pregnancy-0). Long-term, women with gestational diabetes are at risk for the subsequent development of type-2 diabetes (56).

## World-Wide Increase in Obesity, Insulin-Resistance, and Gestational Diabetes

### Obesity Epidemic

Over the past 20 years there has been a significant increase world-wide in obesity, type-2 diabetes, and gestational diabetes (59). Contributors include adoption of a Western diet, high in calories and low in nutritionals (1), lack of access to healthy foods (https://www.ncbi.nlm.nih.gov/pubmed/25032337) (2), and adoption of a sedentary lifestyle.

The National Health and Nutrition Examination Surveys (NHANES) study reported in 2014 that 40.4% of adult women were obese (Body Mass Index (BMI) ≥30 kg/m^2^) (60, 61). Between 2005 and 2014, there was an increased prevalence of obesity (from 35.3 to 40.4%) and severe obesity (from 7.4 to 9.9%) in women (60, 61) but no statistically significant increase was observed in men (60, 61). Obesity has steadily increased in adolescents 12–19 years of age; severe obesity increased in adolescents from 2.6% in 1988–1994 to 9.1% in 2013–2014 (60–62).

According to the United States Center for Disease Control (CDC), in 2015, 30.3 million people (12.2% all U.S. adults) across the United States are estimated to have type-2 diabetes (https://www.cdc.gov/diabetes/data/statistics/statistics-report.html); this total included 7.2 million (23.8% of total) who were not aware. Compared to non-Hispanic whites, the age-adjusted prevalence of diagnosed and undiagnosed diabetes was higher among Asians, non-Hispanic Blacks, and Hispanics during 2011–2014. The world-wide incidence of diabetes, insulin-resistance, and gestational diabetes is difficult to estimate as many cases are undiagnosed and unreported [see Ogurtsova et al., 2017 for a full review (63)]. However, it is clear that the incidence is increasing. According to Ogurtsova et al., in 2015 it was estimated that world-wide there were 415 million (CI: 340– 536 million) people with type-2 diabetes (63). The epidemic of type-2 diabetes disproportionately was felt by people living in poverty; 75% of individuals with type-2 diabetes were living in low- and middle-income countries. By 2040, the number of individuals living with type-2 diabetes is predicted to rise to 642 million world-wide (CI: 521–829 million) (63).

Achieving a healthy energy balance requires physical activity. Over the past 20 years, there has been a significant reduction in the number of hours individuals in both the United States and world-wide engage in exercise. This decrease in exercise has had important consequences; the decrease has worsened metabolic health and increased insulin-resistance of skeletal muscle.

Social inequities have contributed to a decrease in exercise in African-Americans/Blacks and Hispanic/Latinos. Access to public parks, public pools, and green space is much lower in African-American/Black and Latino/Hispanic neighborhoods. Sidewalks in African-American/Black neighborhoods are likely to be in poor condition (https://www.cdc.gov/mmwr/preview/mmwrhtml/ss6304a1.htm). The threat of violence strongly affects the willingness of mothers to let their children play outdoors (http://www.nrpa.org/publications-research/research-papers/archive/) (64). African-American/Black children in neighborhoods that lack access to parks, playgrounds, and recreation centers have a 20–45% greater risk of becoming overweight (https://theblackdetour.com/the-obesity-crisis-in-black-america/).

As the incidence of obesity and type-2 diabetes continues to rise, women of childbearing age are at increased risk for insulin-resistance and gestational diabetes. The CDC reported in 2014 that 29.3% of women in the United States had insulin-resistance; only 13.3% were aware of having insulin-resistance (https://www.cdc.gov/diabetes/data/statistics/statistics-report.html). The incidence of insulin-resistance was highest in Asians (35.7%) followed by non-Hispanic Blacks (36.3%), Hispanics (31.7%), and non-Hispanic Whites (31.5%) (https://www.cdc.gov/diabetes/data/statistics/statistics-report.html).

## Hyperinsulinemia–Impact on Organs, Organelles, and Specific Tissue-Types

### General Organ Effects

The majority of mammalian organs are impacted by hyperinsulinemia [Figure 3B; reviewed in Haeusler et al. (45)]. In the liver, hyperinsulinemia promotes dyslipidemia, and promotes the development fatty liver. In the brain, hyperinsulinemia stimulates appetite, increasing caloric intake, weight gain, and worsening of hyperinsulinemia (45). In skeletal muscle, hyperinsulinemia and insulin-resistance promotes decreased glucose uptake, increases fatigue, decreases physical activity, and subsequently increases insulin-resistance of muscle tissue (45). In adipose tissue, hyperinsulinemia increases lipid accumulation and promotes inflammation (45). In blood vessels and the kidney, insulin promotes damage to endothelial cells and renal dysfunction due to increased nitric oxide synthesis, increased production of reactive oxygen species, and decreased cell adhesion/increased mobility (45).

### Organelles

While insulin signaling has been extensively studied, there remain many areas of active investigation. For a comprehensive review of insulin-signaling see Haeusler et al. (45).

#### Mitochondria

**Figure 4:**
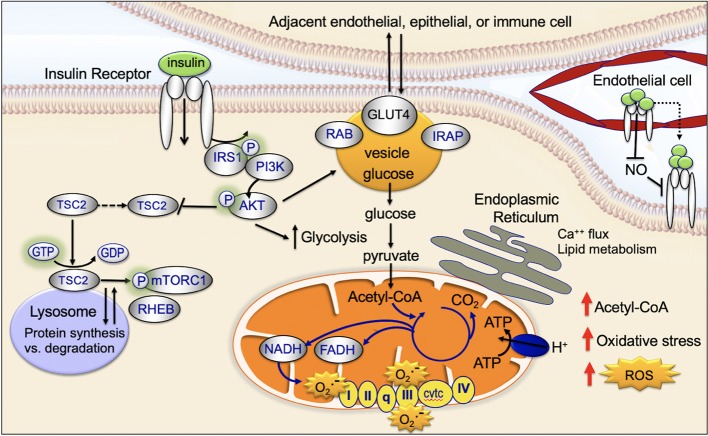
Impact of insulin signaling on subcellular components.

Mitochondria are key targets of insulin signaling (Figure 4). When insulin binds to the insulin receptor, there is downstream activation of IRS1/PI3K/AKT. Activation of AKT increases recruitment of the GLUT4 receptor to the plasma membrane, increases glucose uptake, stimulates glycolysis, and drives the TCA cycle and ATP production (45).

#### Lysosomes

Insulin binds to the cell surface insulin receptor, activating IRS and downstream PI3K/AKT/mTOR-network signaling (Figure 4). The mTORC1 complex is made up of the mTOR, Raptor, PRAS40, mLST8, and DEPTOR. mTORC1 activates key downstream regulatory proteins such as 4EBP1 and S6K1. mTORC1 is activated in the lysosome (45, 65). At the lysosomal membrane, mTORC1 interacts with RHEB. RHEB activates mTORC1 but only if insulin signaling is activated. When insulin signaling is not active, RHEB is bound to the TSC protein complex and is inactive (45, 66). GDP-bound TSC2 binds RHEB and inactivates it; GTP-bound TSC2 releases RHEB and allows it to be activated. Following insulin stimulation, AKT phosphorylation causes TSC2-bound GDP to be converted to GTP, allowing RHEB to be released and activated (45, 66, 67). AKT-mediated phosphorylation has been recently shown to promote dissociation of TSC2 from the lysosome, dissociation of RHEB, and activation of mTORC (45, 68).

#### Endoplasmic Reticulum

Mitochondria physically associate with the endoplasmic reticulum (mitochondrial associated membrane) (45). This association promotes transfer of calcium and lipids between the two organelles (69). There is emerging evidence the mitochondrial associated membranes are an important target site for insulin signaling (Figure 4) (70). The mTORC2 complex is composed of a group of proteins including mTOR, Rictor, mLST8, and mSIN1; mTORC2 activates downstream, FOXO and promotes apoptosis resistance (45). Insulin causes activated AKT and the mTORC2 complex to localize to the mitochondrial associated membranes (45, 71). An increase in mitochondrial associated membrane contacts has been recently shown to dysregulate insulin signaling and glucose metabolism (72).

### Cell-Trafficking

After insulin binds to the insulin receptor, the insulin receptor is activated and then internalized within clathrin-coated vesicles (early endosomes; Figure 4) (45). Within these vesicles, insulin and the insulin receptor remain active for signaling and vesicles colocalize with downstream signaling targets (45, 73, 74). Recent studies provide evidence that endosomal insulin receptor signaling plays a role in the mitogenic but not metabolic effects of the insulin receptor (45, 73, 74).

The glucose transporter, GLUT4, is transported between the intracellular space and the plasma membrane (Figure 4). This transport plays a key role in regulating glucose uptake, particularly in adipose cells and muscle (45). In the absence of insulin-signaling, GLUT4 is present in the intracellular space. Insulin-signaling and downstream AKT-activation promotes translocation of GLUT4 to the plasma membrane; at the plasma membrane GLUT4 transports glucose into the cell (75, 76). When insulin-signaling is no longer active GLUT4 returns to the intracellular space (75, 77).

### Key Insulin-Tissue Targets

#### Skeletal Muscle

**Figure 5:**
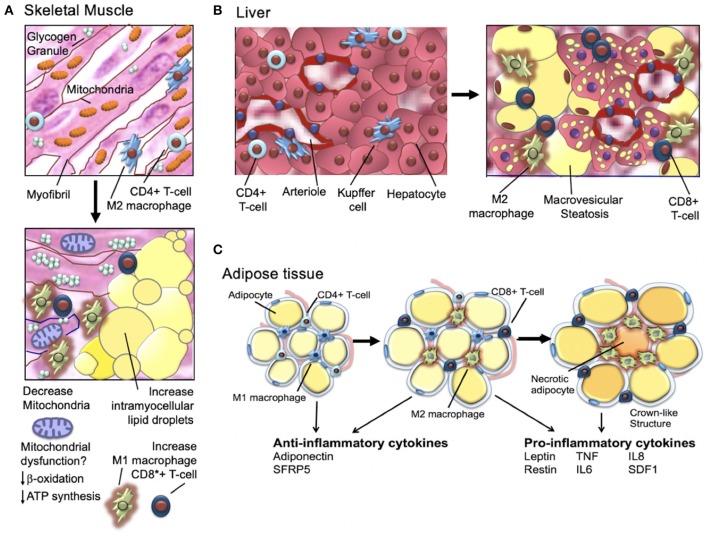
Hyperinsulinemia and **(A)** skeletal muscle, **(B)** liver, and **(C)** adipose tissue.

It has been long known that obese individuals, type-2 diabetics, and individuals who are insulin-resistant have skeletal muscle mitochondrial-defects (Figure 5) (78, 79). Human studies in the 1990's showed that obese and insulin-resistant individuals had reduced muscle oxidative enzyme activity and decreased lipid metabolism compared with lean individuals (80–83); in 2002, it was shown that the skeletal muscle of obese individuals with type-2 diabetes (relative to lean normal controls) exhibited lower mitochondrial oxidoreductase activity and reduced mitochondrial number and size (84–86). In 2003, microarray studies showed mitochondrial muscle biogenesis and oxidative phosphorylation pathways were (1) downregulated in individuals with type-2 diabetes and non-diabetics with a family history of type-2 diabetes and (2) highlighted a key role of the peroxisome proliferator coactivator 1a (PGC1α) as a master regulator of mitochondrial metabolism (78). Subsequent human studies demonstrated similar downregulation of metabolic and mitochondrial pathways in the muscle of individuals with insulin-resistance; key findings were (1) defective expression of muscle mitochondrial genes (mRNA and protein), (2) decreased muscle mitochondrial oxidative enzyme activity, and (3) abnormal mitochondrial size and density [for a full review, see Montgomery and Turner (87)].

#### Adipose Tissue

For a comprehensive review, see Gastaldelli et al. (88). As discussed above, obesity and insulin-resistance are associated with chronic low-grade adipose tissue inflammation that in turn, promotes insulin-resistance and type-2 diabetes (Figure 5) (89). As hypertrophic adipocytes become insulin resistant and lipolytic activity increases, non-esterified fatty acids are shunted away from adipose tissue and deposited in liver and muscle tissue. These changes in the transport of non-esterified fatty acids are associated with increase local production of inflammatory cytokines (89, 90). The increase in inflammatory cytokines further increases lipolysis and promotes activation of macrophages and T cells that, in turn, produce additional high levels of inflammatory cytokines (91). Macrophage infiltration of adipocytes is observed in early insulin-resistance (92). Adipocyte cell death (necrosis) is also a feature of later-stage insulin-resistance (93). Activated macrophages are observed surrounding necrotic adipocytes and together these necrotic adipocytes and surrounding activated macrophages form “crown-like structures” (94). The tissue density of crown-like structures is positively related to adipocyte size, independent of the degree of obesity (93, 94). In a sentinel study, Camastra et al. (95) investigated the impact of bariatric surgery on (1) measured insulin-resistance (insulin clamp), (2) lipolysis (2H5-glycerol infusion), (3) ß-cell glucose-sensitivity (ß-GS, mathematical modeling), and (4) cellular substructure (electron microscopy). Investigators found that pre-surgical subcutaneous fat (SAT) and visceral fat (VAT) demonstrated fibrosis/necrosis, small mitochondria, free interstitial lipids, and a thickened capillary basement membrane (95). Skeletal muscle biopsy demonstrated increased fat infiltration and adipocyte hypertrophy and a reduction in the number and size of mitochondria. Individuals with type-2 diabetes (relative to obese individual without type-2 diabetes) demonstrated impaired ß-GS, intracapillary neutrophils, and higher intramyocellular fat, adipocyte hypertrophy, and crown-like structures in in both VAT and SAT (95). After bariatric surgery, insulin-resistance and lipolysis both decreased. ß-GS improved in individuals with previously diagnosed type-2 diabetes (1) skeletal muscle adipocyte infiltration was reduced, (2) interstitial lipid infiltration was reduced, and (3) the number of smooth muscle cell mitochondria increased (95). The investigators concluded that (1) insulin-resistance improves proportionally to weight loss but remains subnormal and (2) SAT and muscle changes disappear. In individuals with prior type-2 diabetes, after bariatric surgery (1) some VAT pathology persists and (2) beta-cell dysfunction improves but is not normalized (95).

#### Liver

The liver is a key target of insulin and plays an important role in the development of insulin-resistance and type-2 diabetes. Chronic overeating results in the liver (1) losing its ability to regulate and suppress gluconeogenesis but (2) retaining its ability to drive lipogenesis. This dual dysregulation/overproduction of glucose and lipids is characteristic of the hyperglycemia and hyperlipidemia observed in metabolic syndrome and type-2 diabetes (96–98). Chronic hyperglycemia and hyperlipidemia drive non-alcoholic fatty liver disease and atherogenic dyslipidemia (99–101). There has been a great deal of research investigating the molecular mechanism(s) that regulates insulin regulation of gluconeogenesis vs. triglyceride production.

Although the pathophysiology of selective insulin/insulin-receptor signaling remains unclear, it is generally acknowledged that the pathways to insulin regulation of glucose vs. triglyceride production diverge downstream of AKT (96, 102). However, it remains unclear why the divergence would promote, in the face of chronic nutrient overload, the selective inability to suppress gluconeogenesis vs. retention of lipogenesis-regulation. General consensus is that FoxO-signaling plays a key role to account for this dual abnormality (96, 102, 103).

In the liver, a key morphologic feature of chronic nutrient overload, hyperinsulinemia, and lipogenesis is steatosis. Steatosis, or fatty change, is the abnormal retention of lipids within cells. Excess lipid accumulates in hepatocyte vesicles, these vesicles displace the cytoplasm (microvesicular steatosis). When the vesicles are large enough to distort the nucleus, this is called macrovesicular steatosis. Macrovesicular steatosis is important for the development of hepatic fatty liver disease.

#### Wound Healing

Wound healing is impaired in individuals with type-2 diabetes and insulin resistance. Multiple components of wound healing are impaired including (1) neutrophil activation, (2) fibroblast migration and replication, and (3) abnormal angiogenesis. Insulin regulates VEGF expression and hyperinsulinemia is linked to decreased VEGF production (104–108). Reduction in VEGF production and efficacy is linked to increased oxidative stress and hypoxia (109–113). In addition, insulin activates IRS-1 signaling. IRS-1 has also been shown to similarly impair wound healing (114).

### Cancer Processes

#### Glycolysis

**Figure 6:**
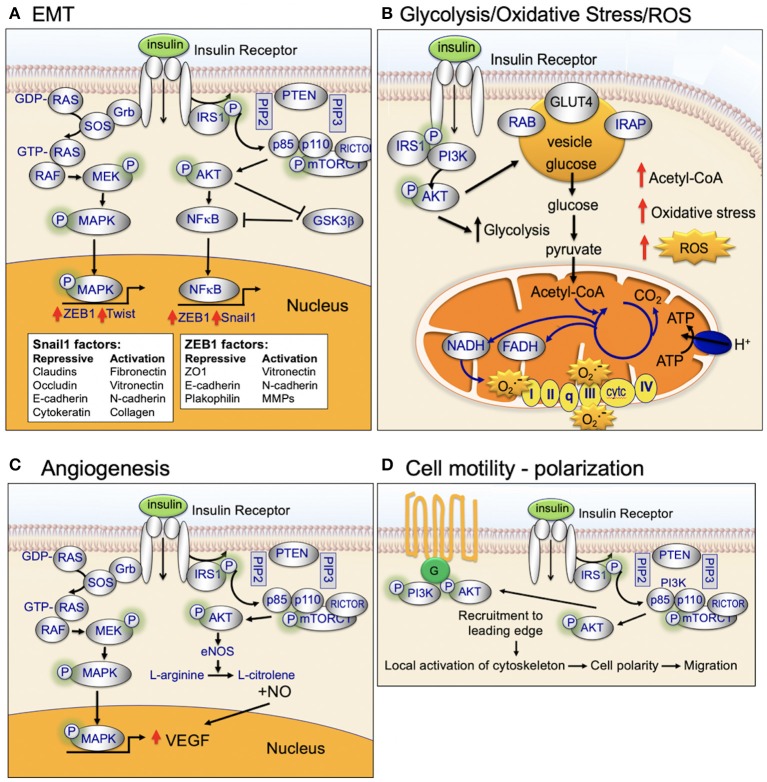
Insulin-signaling in **(A)** EMT, **(B)** Glycolysis, oxidative stress, and ROS, **(C)** Angiogenesis, and **(D)** Cell motility and polarization.

The PI3K/AKT/mTOR-signaling pathway is a well-established regulator of central glucose metabolism and aerobic glycolysis (Figure 6) (115–117). Aggressive cancer cells are known to become glucose dependent and generate a larger proportion of their energy via aerobic glycolysis (Warburg effect) as opposed to mitochondrial oxidative phosphorylation (TCA cycle) (118). The Warburg effect directly contributes to the aggressive biology of cancers by increasing glycolysis/glucose uptake, which supplies anabolic precursors for rapid growth and promotes mitochondrial dysfunction that leads to apoptosis-resistance.

#### Oxidative Stress

Mitochondria are the major sites of cellular reactive oxygen species (ROS) production (Figure 4) (45). Mitochondrial ROS production primarily occurs at complex I (NADH CoQ reductase) and complex III (bc1 complex) (45). Increased ROS production occurs when excess electrons are provided to the mitochondrial respiratory chain: (1) excess electrons are transferred to oxygen and (2) oxygen is converted to superoxide and then to hydrogen peroxide (45). ROS production occurs when the TCA-driven/mitochondrial electron transport-driven proton gradient is high and oxygen consumption/ATP demand is low (45). High calorie intake combined with lack of exercise can (1) increase TCA-driven/mitochondrial electron transport, (2) reduce demand for ATP, and (3) increase ROS production. High levels of ROS damages DNA, proteins, and the lipid bilayer (119, 120).

High ROS production plays an important role in cancer initiation and progression (121). ROS activates pro-oncogenic signaling pathways that play a key role in aggressive breast cancer biology, include RAS, c-MYC, and Wnt/beta-catenin. High ROS production during cancer initiation is linked to high mitochondrial respiration/dysfunction and low coupling-efficiency of the mitochondrial electron transport chain (122). Cancer cells maintain their high energy levels through high aerobic glycolysis (Warburg effect; metabolic switch). This metabolic switch is required for the cancer cells to adapt to the hypoxic tumor microenvironment (123).

#### Cell Motility

Insulin activates both MAPK (RAS/RAF/mitogen activated protein kinase) and PI3K/AKT-signaling (Figure 2). This dual-pathway activation is important for insulin's ability to promote cellular motility and cancer cell invasion (Figure 6).

MAPK-signaling plays a key role in motility and invasion. Under nutrient-rich circumstances, IGF-1 binds to the IGF-1 receptor (IGF-1R) leading to downstream phosphorylation-cascades that activates RAS, RAF, ERK, and MAPK (Figure 2) (124).

Insulin activates AKT. AKT exhibits differential cellular localization in stationary vs. motile cells. Stationary epithelial cells display apical–basal polarity and exhibit an even distribution of basal level of phosphorylated AKT on the plasma membrane, overlapping with cortical actin filaments (125). Twist promotes cell motility through cleavage of intercellular junctions and alterations in cell polarity (apical–basal to front–rear polarity) (126). During Twist-regulated mobility, activated AKT colocalizes with strengthened actin bundles at the leading edge of mobile cells (125). Activated AKT at the leading edge participates in (1) regulation of cell polarity, (2) reorganization of the cytoskeleton, (3) contraction of the cellular body, and (4) thereby promotes cell migration (125).

There is emerging evidence that AKT activation promotes cell motility through direct modulation of cytoskeleton-proteins, including actin and vimentin. Cell motility requires the dynamic remodeling of the cytoskeleton resulting in changes in cell morphology and polarity. Actin has been shown to preferentially bind to phosphorylated AKT at pseudopodia with enhanced bundles (127, 128). The AKT phosphorylation enhancer (APE) protein, or girders of actin filaments (girdin), is an actin-binding protein that maintains the integrity of actin filaments. APE/girdin has been shown to regulate the actin cytoskeleton at the leading edge of migrating cells (125). Depletion of APE/girdin destabilizes actin bundles and depletes actin stress fibers, resulting in loss of directional migration (125). The cytoskeletal protein vimentin is also regulated by AKT1. Phosphorylated AKT1 phosphorylates vimentin at serine-39 (vimentin-pSer39) (129); vimentin-pSer39 is protected from degradation and has been shown to promote invasion (116).

Rho GTPases regulate cell cytoskeleton organization, migration, transcription, and proliferation (130). Rho proteins belong to the RAS superfamily and are activated when bound to GTP (130). Rho controls the stress fibers and focal adhesion formation. Rho downstream proteins Rac and Cdc42, regulate membrane ruffling and filopodium formation, respectively (130). Rho/Rac/Cdc42-signaing participates in dynamic reorganization of the actin cytoskeleton and promotes cell motility (130). Insulin activates AKT which, in turn, activates Rac signaling and promotes cellular motility and invasion (125, 131).

#### Epithelial to Mesenchymal Transition (EMT)

EMT is a cellular process critical for normal embryonic organogenesis. Dysregulation of EMT is frequently observed in biologically aggressive cancers, including breast cancer, and promotes invasion, metastasis, and poor survival (131). Key transcription factors that promote dysregulated EMT during cancer initiation and progression include Snail, Twist, and Zeb. Over the past 10 years, there have been many studies investigating the link between activated AKT and EMT-transcription factor signaling (Figure 6).

Snail is a transcription factor that promotes EMT, migration, and invasion. Snail is phosphorylated by GSK3β in normal epithelial cells. Snail is expressed at low levels in normal cells. Under normal cellular homeostasis, GSK3β phosphorylates Snail and thereby promotes continuous degradation (132, 133). However, in the presence of insulin signaling, active AKT phosphorylates GSK3β, inactivating GSK3β, and stabilizes Snail. Activated AKT2 has been shown to directly stabilize Snail1 binding to the *CDH1* (E-cadherin gene) promoter through direct protein–protein interaction (134). A second EMT transcription factor, Twist, has been shown to bind directly to, and activate, AKT2 transcription in breast cancer cells (135, 136). AKT has been shown to phosphorylate and activate Twist1 (137–139). Recent data also provides evidence that the polycomb group protein, Bmi-1, is a downstream target of Twist1 and a key regulator of EMT and cancer metastasis (140). AKT has been shown to directly phosphorylate Bmi-1 (141). Taken together, these studies demonstrate an important role for insulin and activated AKT in regulating and interaction with EMT transcription factors and promoting EMT and invasion (125).

#### Hyperinsulinemia Promotes Tissue Inflammation

Insulin activates PI3K/AKT-signaling and downstream NFκB. NFκB is a major regulator of tissue inflammation. NFκB increases production of (1) inflammatory cytokines including interleukin-1 (IL-1), IL-2, IL-6, IL-8, IL-12, and TNF-α and (2) chemokines MCP-1, MCP-2, CXCL1, CXCL10, and RANTES (142). Together, these highly potent cytokines and chemokines promote tissue inflammation and angiogenesis. NFκB also plays an important role in immune cell activation, differentiation, and macrophage switching from an M2 to M1 phenotype (142). NFκB plays a central role in regulating T-cell differentiation. Upon activation, CD4+ T-cells differentiate into effector T-cells including Th1, Th2, and Th17 cells; Th1 and Th17 are inflammatory T-cells and secrete interferon-gamma (INF-γ) (142).

Hyperinsulinemia also promotes macrophage switching and macrophage-mediated inflammation (143). Activation of PI3K/AKT- and NFκB-signaling as well as generalized tissue inflammation promotes an increase in the ratio of M2 (anti-inflammatory) to M1 (pro-inflammatory) macrophages (144). This increase in the ratio of M2/M1 macrophages results in an increase in tissue inflammation, particularly in adipose tissue (144). There is also evidence that this increase further increases insulin resistance of muscle. There is also evidence that insulin has complex effects on macrophage polarization and function [for further discussion of this evolving area of research, see Kraakman et al. (144)].

#### Inflammation Promotes Insulin-Resistance

As early as the 1800's, physicians discovered that high dose (now known to be anti-inflammatory) salicylates (5.0–7.5 g/d) reduced glycosuria in diabetic patients (78, 79, 145, 146). Subsequently, Hotamisligil and Karasik (147–149) first showed that that the overproduction of the proinflammatory cytokine tumor necrosis factor-alpha (TNF-α) by adipocyte tissue could induce insulin-resistance. The concept that a substance overproduced by adipose could regulate glucose tolerance and metabolism was groundbreaking (78, 150, 151). Subsequent research identified adipose tissue as a key producer of inflammatory cytokines and chemokines that included, leptin, IL-6, resistin, monocyte chemoattractant protein-1 (MCP-1), PAI-1, angiotensinogen, visfatin, retinol-binding protein-4, and serum amyloid A (SAA) (78, 152–156).

Leptin and adiponectin are true adipokines that appear to be produced exclusively by adipocytes; leptin expression increases with increased adiposity; adiponectin expression decreases with increased adiposity (78, 157). The inflammatory cytokines TNF-α, IL-6, MCP-1, visfatin, and PAI-1 are produced in both adipocytes and activated macrophages found in obese and insulin-resistant individuals (78). Resistin production is less well-understood and includes macrophages in humans but both adipocytes and macrophages in rodents (78, 154). In obese individuals, TNF-α, IL-6, and resistin promote subacute inflammation and MCP-1 plays a role in recruiting macrophages to adipose tissue (78). Together, these inflammatory cytokines and chemokines activate intracellular pathways that promote insulin-resistance and, ultimately, type-2 diabetes (78).

#### Inflammation Promotes Atherosclerosis

Hyperinsulinemia and tissue inflammation is also closely linked to atherosclerosis (78). In obese individuals TNF-α, IL-6, and resistin promote subacute vascular inflammation associated with upregulation of cell adhesion molecules P- and E-selectin, ICAM-1, and VCAM-1, that (1) act to localize circulating immune cells, (78, 158, 159) and (2) increase local production of inflammatory cytokines and chemokines that include MCP-1 and macrophage inflammatory protein-1α (MIP-1α), MIP-1β, MIP-2, and MIP-3α (78, 160–162). T-cells are activated in this inflammatory pro-atherosclerosis microenvironment and produce IFN-γ and lymphotoxin. Activated macrophages, endothelial cells, and smooth muscle cells produce TNF-α (78, 160, 161). Together, these processes increase local production of IL-6 in the atheroma (162, 163). Engagement of CD40 and CD40 ligand increases local production of matrix-metalloproteases (MMPs) (164). MMPs in turn break down collagen and promote thrombosis (164, 165).

#### Angiogenesis

Angiogenesis is a key process in cancer initiation and metastasis. Endothelial cells and pericytes are two key cell types that participate in vessel formation and maturation and both endothelial cells and pericytes express the insulin receptor (166). Insulin, through insulin-receptor network signaling regulates endothelial cell migration, proliferation, and production of VEGF vascular growth factors (Figure 6) (166). Emerging evidence highlights an important role for insulin signaling in deregulation of normal angiogenesis [see Escudero et al. for a review of this topic (166)]. Insulin activates MAPK leading to increased endothelial cell survival and proliferation. In addition, insulin activation of PI3K/AKT signaling promotes increased nitric oxide release. Nitric oxide increases endothelial survival, migration, proliferation, and vascular permeability (166).

## BMI Does Not Always Reflect Metabolic Health

### BMI—An Inexact Measure

The most frequently used measure of adiposity is BMI ≥30 kg/m^2^ (Centers for Disease Control and Prevention, https://www.cdc.gov/obesity/adult/defining.html, accessed 4/3/2017). While BMI is a commonly used measure, the appropriateness of BMI as a phenotypic marker of adiposity across populations differing in race and ethnicity is highly controversial. BMI is an inexact measure of metabolic dysfunction; comparing BMI between individuals of different races and ethnicities has inherent problems (167, 168).

BMI is a measure of mass (kilograms or pounds) per area (meters-squared or inches-squared; BMI is not a direct measure of obesity. Muscle weighs more than fat; consequently lean, muscular individuals can be misclassified overweight or obese when BMI is used as the sole measure of adiposity (169, 170). Furthermore, the relationship between BMI and adiposity varies significantly between different races and ethnic groups. The relationship(s) between body shape/composition and disease is an inexact science and only beginning to be understood (59, 169, 170). It is well-recognized, however, that African-Americans/Blacks have higher muscle mass than non-Hispanic Whites and Asians (59).

There is a second problem with using BMI as a surrogate measure for metabolic health. The BMI threshold for type-2 diabetes risk markedly varies in individuals of different races and ethnicities (59). In a large multiethnic cohort study, for an equivalent incidence rate of type-2 diabetes conferred by a BMI of 30 kg/m^2^ in non-Hispanic Whites, the BMI was found to be 26 kg/m^2^ in African-Americans/Blacks, 25 kg/m^2^ in Chinese-Americans, and 24 kg/m^2^ in South Asians (171, 172). Consequently, a non-Hispanic White woman with a BMI of 25 mg/m^2^ is likely to be metabolically healthy, while an Asian woman with the same BMI has a high likelihood of being pre-diabetic or even diabetic (171, 172). Taken together, these studies provide evidence that body composition and/or the insulin-sensitivity of peripheral muscle tissue could play a role in determining metabolic health of individuals of diverse races and ethnicities.

#### Metabolically Unhealthy Normal-Weight and Metabolically Healthy Obese Individuals

Over the past 20 years, there is increasing recognition that obese individuals (BMI >30 kg/m^2^) can have normal metabolic profiles, “metabolically healthy obese” (173–176). While the precise definition of “metabolically healthy obese” varies, the generally accepted definition is individuals with a BMI > 30 kg/m^2^ who do not have insulin-resistance, type-2 diabetes, dyslipidemia, or hypertension (175–178).

In contrast to individuals who are obese but metabolically healthy, there are also individuals with a normal BMI (BMI < 25 kg/m^2^) who have abnormal metabolic profiles and increased cardiovascular risk. These individuals were first described by Ruderman et al. as hyperinsulinemic, insulin resistant, hypertriglyceridemic, and predisposed to subsequent development of type 2 diabetes mellitus and coronary artery disease (179). Currently there is no consistent definition, but the generally accepted definition includes (1) BMI < 25 kg/m^2^, (2) metabolic abnormalities that include insulin resistance, hypertriglyceridemia, (3) abdominal fat distribution, and (4) elevated blood pressure (179). Most studies set the cutoff as three or more metabolic derangements to fulfill “metabolically unhealthy” definition.

#### Racial and Ethnic Differences in Insulin-Sensitivity

Peripheral muscle tissue is the major determinant of insulin-sensitivity; this is why exercise is thought to have such a key impact on improving insulin-sensitivity and metabolic health in insulin resistant individuals. It is known that that African-Americans/Blacks have lower insulin sensitivity of their peripheral muscles than non-Hispanic White women (59, 180). Lower peripheral insulin sensitivity in African-American/Black women compared to non-Hispanic White women could account for these differences and, thus, make is difficult to determine “metabolically healthy” BMI cut off points (172).

#### Asians and Underdiagnosis of Insulin-Resistance

Asians have a disproportionately increased incidence of diabetes mellitus relative to other racial and ethnic groups, in the United States and worldwide. By the International Diabetes Federation estimates for 2017, China and India have the largest number of people 20–79 years old with diabetes at 114.4 and 72.9 million, respectively, compared to the next highest in the United States at 30.2 million (IDF Diabetes Atlas, 8th edition 2017, https://www.idf.org/e-library/epidemiology-research/diabetes-atlas/134-idf-diabetes-atlas-8th-edition.html). Western influences on lifestyle including diet and physical activity likely contribute this burgeoning problem in China, India, and other parts of Asia as well as in the United States for Asian-Americans.

Asians tend to have increased adiposity, and in particular higher visceral fat, relative to Caucasians and other non-Asians within the same range of body mass index (BMI). For a given BMI, Asian-Americans have a higher likelihood of developing diabetes compared to non-Hispanic whites (181). BMI does not accurately screen for visceral adiposity, such that standard BMI cut-off points based on non-Hispanic Caucasian populations underestimate obesity-related health risks in Asians (182). The international BMI cut-off points of the World Health Organization are not applicable to Asians for risk assessment and potential intervention to prevent and treat obesity-related diseases such as diabetes. In considering the different associations of BMI, body fat, and disease in Asians vs. Caucasians, coupled with highly heterogeneous Asian subpopulations, the WHO expert consultation of 2002 did not recommend a change in international standard cut-off points for BMI and instead identified public health action points along the BMI continuum for guidance in tailoring BMI cut-off points for a specific country (23). For Asian-Americans, the optimal BMI cut-off point may vary for different subpopulations (183). Based on consolidated data from multiple population and community-based studies of Asian-Americans, screening for BMI ≥ 25 kg/m^2^ would have missed 36% of diabetics; screening at a lower BMI ≥ 23 kg/m^2^ increased the sensitivity from 63.7 to 84.7% and missed 15% of diabetic Asian-Americans (184). For identifying Asian-Americans to screen for undiagnosed diabetes, Araneta et al. therefore suggest a BMI cut-off point of ≥23 and <25 kg/m^2^ (184). In 2015, the American Diabetes Association revised the BMI criteria for diabetes screening from ≥25 to ≥23 kg/m^2^ for all Asian-Americans <45 years of age; possibly a lower BMI cut-off is needed to screen for pre-diabetes (185). Notably, Asian-Americans are the least likely racial and ethnic group to undergo recommended diabetes screening, with 34% lower adjusted odds relative to non-Hispanic white Americans in the 2012–2014 Behavioral Risk Factor Surveillance System database (186).

Reduction in insulin secretory function in Asians relative to insulin resistance may contribute to increased risk of diabetes. In studies of Japanese patients, impaired insulin secretion seems the main driver of diabetes and prediabetes, with lesser role for insulin resistance (187, 188). Interestingly, in a study of pancreatic tissue obtained from diabetic and non-diabetic Korean patients, non-obese patients with type 2 diabetes had ~50% decreased volume of β-islet cells compared to BMI-matched patients without diabetes, raising the possibility that lower BMI and smaller β-islet cell mass might underlie the pathogenesis of diabetes in non-obese Koreans (189).

Standard screening tests may miss diagnoses of diabetes in Asians. In a study of 1214 Asian-American participants without prior diabetes diagnosis, HgbA1c failed to diagnose ~50% of Asian-Americans with diabetes; 44% of participants were diagnosed by post-prandial glucose levels (184).

## Epidemiology Studies Supporting the Link Between Insulin and Breast Cancer

The majority of studies have focused on investigating the potential link between BMI and breast cancer subtypes. More recently, studies have investigated the potential link between parameters of metabolic health such as insulin and HgbA1c. The American Diabetes Association and the American Cancer Society issued a 2010 consensus report stating that type-2 diabetes was associate with cancers, including breast (35). A recent meta-analysis of 23 studies found diabetes to be associated with an increased mortality hazard ratio (HR) of 1.41 (95% CI 1.28–1.55) in individuals with cancer, including breast (36). A meta-analysis of Israeli non-diabetic women followed for over 35 years, investigated the potential link between basal- and fasting-insulin levels and risk for breast, colon/rectal, and bladder cancer (37). Basal insulin level was not significantly associated with cancer of the breast, colon/rectum, or bladder). Fasting insulin in the upper quartile conferred a 37% increased risk for total mortality among cancer patients, adjusting for age and ethnic origin (95% CI 0.94–2.00, *P* = 0.097) compared with that of the lower quartiles (37). This long-term cohort study may suggest a role for basal elevated insulin levels, mainly as a negative predictor in cancer prognosis (37). This is consistent with studies showing that individuals with type-2 diabetes who are treated with insulin have an increased risk for malignancies, including breast cancer (38).

## Insulin-Signaling and Breast Cancer Biology

As discussed above (section Complex Relationship Between Obesity and Breast Cancer) BMI and obesity have not been consistently associated with increased risk with premenopausal TNBC. However, as also discussed above (section BMI Does not Always Reflect Metabolic Health), BMI and obesity do not consistently predict metabolic health; individuals can be normal weight and metabolically unhealthy; conversely individuals can be obese and metabolically healthy. Recent studies provide evidence that metabolic health (rather than BMI) may be a better predictor of breast cancer risk (Table 1B). Combined metabolic dysfunction and obesity in post-menopausal women have been shown to be a stronger predictor of breast cancer risk than obesity alone (39). In the Sister Study, women with BMI <25 kg/m^2^ and ≥1 metabolic abnormality (metabolically unhealthy; normal weight) vs. women BMI <25 kg/m^2^ and no metabolic abnormality (metabolically healthy; normal weight) had increased risk of post-menopausal breast cancer (40). In a third study, post-menopausal women with normal BMI, high body adipose tissue was associated with increased (1) metabolic dysfunction and circulating inflammatory factors and (2) risk of invasive breast cancer (41). In the Women's Health Initiative Observational Study, hyperinsulinemia was found to be an independent risk factor for post-menopausal breast cancer (42). These studies in post-menopausal women provide evidence that metabolic dysfunction, rather than BMI may be a better predictor of breast cancer risk; additional studies are needed to evaluate the potential relationship between metabolic dysfunction, insulin, and breast cancer risk in premenopausal women. Below are mechanisms that could account for a potential link between hyperinsulinemia and breast cancer risk.

### PI3K/AKT/mTOR

As detailed above, insulin signaling activates PI3K/AKT/mTOR; PI3K/AKT/mTOR signaling promotes proliferation, apoptosis-resistance, and invasion (125, 190–194). TNBCs are ER-/PR- and HER2-not amplified. TNBC occur most frequently in BRCA1 mutation carriers and young African-American/Black women and frequently carry a poor prognosis (59). Basal-type breast cancers are a subtype of TNBC that are identified by specific gene expression patterning (59). In basal-type breast cancer, the Tumor Genome Atlas showed in basal-type breast cancer that the PI3K/AKT/mTOR-signaling pathway was frequently activated (192, 194). AKT activation (over expression of phosphorylated-AKT) predicts poor prognosis in women with breast cancers, but not all studies show a consistent association (193). A recent systematic review tested for the association of phospho-AKT expression in breast cancer with overall survival and disease-free survival (193). In this systematic review, 33 studies (9,836 women) were evaluated from three diverse electronic databases: (1) PubMed, (2) EMBASE, and (3) Chinese Biomedical. In women with breast cancer, overexpression of phosphorylated AKT was associated with worse overall survival and disease-free survival, respectively, 1.52 (95% CI: 1.29–1.78) and 1.28 (95% CI: 1.13–1.45) (193). Worse overall survival was predicted in all breast cancer subgroups (193). Taken together, these studies provide evidence that activation of AKT-signaling is an adverse prognostic factor in breast cancer and support the rational for normalizing insulin-driven PI3K/AKT/mTOR-signaling pathway in women with breast cancer and woman at risk for breast cancer.

### Glycolysis

Biologically aggressive breast cancers, particularly TNBC and poor prognosis luminal B breast cancers (ER+/HER2-wt or -amplified, Ki67 ≥14%) frequently exhibit high glucose consumption and aerobic glycolysis (195, 196). Aggressive cancer cells are known to become glucose dependent and generate a larger proportion of their energy via aerobic glycolysis (Warburg effect) as opposed to mitochondrial oxidative phosphorylation (TCA cycle) (197). The Warburg effect directly contributes to the aggressive biology of cancers by increasing glycolysis/glucose uptake, which supplies anabolic precursors for rapid growth and promotes mitochondrial dysfunction that leads to apoptosis-resistance. Dysregulation of PI3K/AKT/mTOR-signaling is a regulator of aerobic glycolysis (194, 197–199) and provides scientific rational for controlling insulin-activation of PI3K/AKT/mTOR in both women with breast cancer and at-risk women.

### Immune Cell Switching, and a Pro-tumorigenic Tissue Microenvironment

As discussed above, there is emerging evidence that insulin signaling plays a role in macrophage switching during cancer initiation and progression. In addition, there is increasing evidence that T-cell subsets and macrophages can promote the aggressive biology of TNBC. T-cells and macrophages can either inhibit or promote tumorigenesis. Classically activated macrophages (M1-type) are regulated by T_H_1 cytokines (e.g., IFNγ or TNFα); M1 macrophages possess enhanced cytotoxic activity and are anti-tumorigenic (200), however, when tissue is exposed to inflammatory cytokines (e.g., leptin, IL-6, IL-8, IL-12, CCL2, and IL-1β), there is a switch from M1 macrophages to alternatively activated macrophage (M2-type) (59, 200, 201). In non-cancerous tissue, M2 macrophages play a key role in tissue repair. However, there is increasing evidence that activated M2 macrophages promote the aggressive biology of TNBC. M2 macrophages are found in high numbers in stroma of TNBC (59, 202). M2 macrophages secrete epithelial growth factor (EGF) and tumor growth factor-beta (TGFβ). Poor prognosis TNBC is characterized by activation of EGF- and TGFβ-signaling. EGF and TGFβ both promote invasion, metastasis, and progenitor-cell turnover. Emerging evidence shows that EGF and TGFβ-signaling contributes to a pro-tumorigenic microenvironment that contributes to initiation and progression of TNBC (203, 204). Taken together, these observations underscore a potential mechanistic link between insulin, M2 macrophage production of EGF and TGFβ, and aggressive TNBC biology.

## Treatment of Insulin-Resistance and Biology of Metformin

Treatment of obesity-related end-organ failure (type-2 diabetes) is expensive. However, much of our efforts in preventing diabetes through diet and exercise have not been successful and type-2 diabetes is frequently not diagnosed until complications occur. This has led to a call for early screening and treatment of individuals at high-risk for type-2 diabetes.

### Metformin

Metformin (1,1-dimethylbiguanide hydrochloride) is a well-tolerated oral biguanide hypoglycemic agent that is prescribed to over 120 million type-2 diabetic patients worldwide (https://www.drugs.com/monograph/metformin-hydrochloride.html). Metformin is prescribed for first-line treatment of type-2 diabetes (https://www.drugs.com/monograph/metformin-hydrochloride.html) (56, 205–207) and is also approved for treatment of polycystic ovary syndrome and gestational diabetes (https://www.drugs.com/monograph/metformin-hydrochloride.html) (207). Metformin is generally well-tolerated (208). Common side effects include diarrhea, nausea, and epigastric pain (https://www.drugs.com/monograph/metformin-hydrochloride.html). Metformin inhibits hepatic gluconeogenesis and decreases intestinal absorption of glucose, secondarily decreasing circulating insulin (https://www.accessdata.fda.gov/drugsatfda_docs/label/2006/021748s002lbl.pdf). Metformin is also thought to indirectly increase insulin sensitivity by increasing peripheral glucose uptake and utilization (https://www.accessdata.fda.gov/drugsatfda_docs/label/2006/021748s002lbl.pdf). Given its efficacy and excellent safety profile, metformin is on the World Health's Organization list of essential medicines and has been used for glucose control since the 1960's (http://www.who.int/medicines/publications/essentialmedicines/EML_2015_FINAL_amended_NOV2015.pdf?ua=1).

### Metformin Mechanism of Action

**Figure 7:**
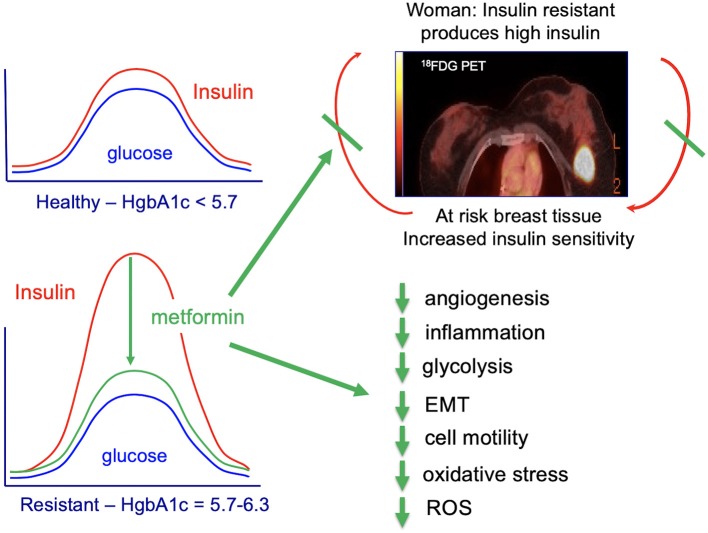
Impact of metformin on cancer processes.

Despite its long history of clinical use, the precise molecular mechanism(s) underlying metformin's insulin-lowering effects, as well as its potential anti-neoplastic potential, are not completely understood. It is well-established that metformin inhibits hepatic gluconeogenesis and secondarily lowers circulating insulin levels (Figure 7) (209). The secondary lowering of insulin by metformin inhibits insulin/IGF-1-signaling and downstream (1) PI3K /AKT/mTOR-network signaling and (2) RAS/RAF/mitogen activated protein kinase (MAPK) (Figure 7) (210, 211). Metformin activation of AMPK (1) inhibits complex-I in the mitochondrial electron transport chain (210, 212, 213), (2) reduces ATP production and increases binding of AMP to AMPK, and (3) increases the substrate affinity of AMPK for LKB1 (214). Metformin activation of AMPK-LKB1 inhibits AKT/mTOR-network signaling leading to downstream inhibition of S6-Kinase (S6K) and 4E binding protein-1 (4EB-1) (210).

### Metformin Prevention of Type-2 Diabetes

There is increasing evidence that metformin, with or without lifestyle modification, in young, high-risk individuals can reverse and prevent type-2 diabetes. In the Diabetes Prevention Study (DPS), metformin was as effective as lifestyle modification in participants <60 years of age (208) and in women with a history of gestational diabetes metformin and intensive lifestyle modification led to a 50% reduction in the incidence of type-2 diabetes.

Several well-controlled studies show that it is possible to prevent type-2 diabetes through a combination of diet, exercise, and metformin. The Diabetes Prevention Program/Diabetes Prevention Program Outcomes Study (DPP/DPPOS) is the largest and longest clinical trial of metformin for the prevention of type-2 diabetes (215, 216). The DPP (1996–2001) was a randomized control trial that followed 3,234 adults from 1996 to 2001 who were, at baseline, at high-risk of developing diabetes. Participants were randomized to receive (1) placebo (*n* = 1,082), (2) metformin (*n* = 1,073) 850 mg twice daily, or (3) intensive lifestyle intervention (*n* = 1,079) (215, 216). The metformin/placebo intervention arm was terminated 1 year ahead of schedule because of demonstrated efficacy (215). The primary outcome was reported at 2.8 years. At the end of the DPP trial, 88% (*n* = 2,776) of the cohort elected to be evaluated in the DPPOS follow-up trial (215, 216).

Study participants in the DPP/DPPOS cohort have over 15 years prospective assessment of the impact of metformin and lifestyle modification on type-2 diabetes, cardiovascular events, and health, economic, and safety outcomes (215, 216). After an average follow-up of 2.8 years, metformin reduced the incidence of diabetes by 31% compared with placebo, with a greater effect in subjects who were obese or had a history of gestational diabetes (56). Analysis of the DPP/DPPOS trial showed that metformin was less effective than lifestyle modification in the DPP/DPPOS, but in obese individuals (body mass index >35 kg/m^2^), metformin was as effective a lifestyle modification (216). For women with a history of gestational diabetes, metformin and intensive lifestyle modification led to an equivalent 50% reduction in diabetes risk (217).

Based on findings from the DPP/DPPOS study, in 2014, the American Association of Diabetes published formal recommendations for prevention of type-2 diabetes (Table 2) (56). Recommendations included: individuals with impaired glucose tolerance or a HgbA1c 5.7–6.4% should be referred to a life-style modification to target 7% weight loss and moderate physical activity (e.g., walking) for 150 min/week. Metformin was recommended for prevention of type-2 diabetes in individuals with impaired glucose tolerance or HgbA1c 5.7–6.4%, especially in individuals with a BMI >35 kg/m^2^ and women with prior gestational diabetes. Treatment of insulin-resistance with metformin makes strong biological and economic sense. Metformin is cheap, safe (used routinely during pregnancy), and effective. Despite the fact that metformin prevention of type-2 diabetes in high-risk individuals is now standard of care in the United States, many clinicians continue to focus on exclusively on treatment of type-2 diabetes and not its prevention.

**Table 2:** American Diabetes Association for prevention of type-2 diabetes [summarized from ref (56)].

**(A) Level of Evidence Description**
**1. Level A**
Clear evidence from well-conducted, generalizable RCTs that are adequately powered, including:
Evidence from a well-conducted multicenter trial
Evidence from a meta-analysis that incorporated quality ratings in the analysis
Compelling non-experimental evidence, i.e., “all or none” rule developed by the Center for Evidence-Based Medicine at the University of Oxford
Supportive evidence from well-conducted RCTs that are adequately powered, including:
Evidence from a well-conducted trial at one or more institutions
Evidence from a meta-analysis that incorporated quality ratings in the analysis
**2. Level B**
Supportive evidence from well-conducted cohort studies
Evidence from a well-conducted prospective cohort study or registry
Evidence from a well-conducted meta-analysis of cohort studies
Supportive evidence from a well-conducted case-control study
**3. Level C**
Supportive evidence from poorly controlled or uncontrolled studies
Evidence from randomized clinical trials with one or more major or three or more minor methodological flaws that could invalidate the results
Evidence from observational studies with high potential for bias (such as case
series with comparison with historical controls)
Evidence from case series or case reports
Conflicting evidence with the weight of evidence supporting the recommendation
**4. Level E**
Expert consensus or clinical experience
**(B) Recommendations and level of evidence**
Patients with impaired glucose tolerance **A**, impaired fasting glucose **E**, or HgbA1c 5.7–6.4% **E** should be referred to a support program targeting weight loss of 7% of body weight and increasing physical activity to at least 150 min/week of moderate activity such as walking.
Follow-up counseling appears to be important for success. **B**
Metformin therapy for prevention of type 2 diabetes may be considered in those with impaired glucose tolerance **A**, impaired fasting glucose **E**, or HgbA1c 5.7–6.4% **E**, especially for those with BMI >35 kg/m^2^, aged,60 years, and women with prior gestational diabetes. **A**
At least annual monitoring for the development of type-2 diabetes in those with prediabetes is suggested. **E**

## Time Points During Breast Cancer Prevention and Treatment Where Insulin Signaling Could be Better Controlled

### Prevention—Current Opportunities

As discussed above, insulin activates many key-signaling pathways and cancer processes that are key for cancer initiation and progression including: EMT, cell migration and mobility, tissue inflammation, ROS production, glycolysis and, perhaps, angiogenesis (Figure 7). Metformin secondarily lowers circulating insulin and the many pro-cancer signaling pathways regulated by PI3K/AKT/mTOR (209). There are two major signaling pathways that are thought to account for metformin's potential anti-cancer activity: (1) AMPK (adenosine monophosphate (AMP)-activated protein kinase)-independent, driven by metformin's ability to secondarily lower serum insulin and (2) AMPK-dependent, regulated by metformin-suppression inhibition of mitochondrial complex-I (complex-I) (209); both pathways converge on mTOR; these actions support the use of metformin for prevention of biologically aggressive breast cancers (209).

Currently there is a lack of effective breast cancer prevention for women who are at risk for TNBC and women who carry germline *BRCA1* mutations. Given that PI3K/AKT/mTOR is a key driver of the aggressive biology of TNBC and metformin inhibits PI3K/AKT/mTOR, there has been interest in testing whether metformin maybe effective for prevention of TNBC in *BRCA1* germline mutation carriers. In addition to targeting PI3K/AKT/mTOR, metformin targets additional signaling networks regulated by AMPK. Metformin-targeting of AMPK for prevention of TNBC in *BRCA1* mutation carriers has good rationale because of the dual signaling networks regulated by both AMPK and BRCA1, include acetyl coenzyme A carboxylase alpha (ACCA), p53, and PTEN (209, 218–220). AMPK regulates the phosphorylation/dephosphorylation cycles of ACCA (209). Given that AMPK and BRCA1 both inactivate ACCA, this provides a molecular mechanism by which metformin might substitute for loss of *BRCA1* tumor suppressive function.

Over the past 10 years, there have been important efforts to repurpose drugs for breast cancer prevention. To this end, metformin is being actively tested for primary and secondary prevention of breast cancer [for a full review, see Heckman-Stoddard et al. (210) (Table 3)]. The largest adjuvant (secondary prevention) trial is NCIC MA.32, a phase III adjuvant breast cancer trial randomizing 3,649 women within 12 months of diagnosis to metformin 850 mg p.o. twice a day (850 mg/day during weeks 1–4) vs. placebo for 5 years (Table 3, NCT01101438) (http://clinicaltrials.gov/ct2/show/study/NCT01101438). The primary endpoint is invasive disease-free survival. Studies testing the impact of metformin in the unaffected breast include a study in women scheduled for a reduction mammoplasty that compares changes in LKB1 and AMPK signaling in women treated with metformin 500 mg twice a day (dose escalated) vs. no treatment (Table 3, ACTRN12610000219088) (http://apps.who.int/trialsearch/trial2.aspx?trialid=ACTRN12610000219088). There are two ongoing larger primary prevention studies one in overweight and obese premenopausal women with high breast density (Table 3, NCT01793948) and one testing metformin 850 mg bid in high-risk premenopausal women with cytologic atypia that allows for inclusion of women with germline *BRCA1* and *BRCA2* mutations (Table 3, NCT01905046) (210). Together, these trials will provide important evidence whether metformin is an effective agent for prevention of breast cancer.

**Table 3:** Adjuvant, secondary prevention, and primary prevention trials utilizing metformin.

**Trial/ClinicalTrials.gov**	**Study design metformin dose**	**Study population**	**Target accrual (planned/evaluable)**	**Primary endpoint**
**(A) Adjuvant and secondary prevention trials** (210)				
Breast Phase II	500 mg tid for 6 months	IBC completed therapy with fasting insulin of ≥45 pmol/L and glucose < 7.0 mmol/L	40/22	Change in insulin levels 22.4% decrease (*p* = 0.024)
Breast Phase I NCT0089788459	500 mg bid for 2–3 weeks	Women < 70 Pre-surgical- IBC T1-4	48/39	2.97% decrease in Ki-67 (*p* = 0.016)
Breast Phase II 2008-004912-10	850 mg/d for 3 days followed by 850 mg bid day 4–28 vs. placebo for 4 weeks prior to surgery	Presurgical-Stage IIII IBC patient not suitable for neoadjuvant therapy	200/196	No overall change in Ki-67 10.5% decrease in Ki-67 if HOMA >2.8 (p for interaction = 0.045)
Breast Phase II 2007-000306-70	500 mg/d for 1 week followed by 1,000 mg/d for 1 week vs. placebo	Stage 1–2 IBC, >1 cm, no history of diabetes	47/39	3.4% decrease in Ki-67 (*p* = 0.02)
Breast Phase 0 NCT0198082360	500 mg am and 1000 mg pm metformin with 80 mg atorvastatin for at least 2 weeks prior to surgery	Histologically confirmed DCIS or IBC who undergo CNB followed by surgery	40	No reduction in Ki-67
Breast Phase II 2006-006236-22	1,000 vs. 1,500 mg/d for 3 months	Post-menopausal with history of IBC and 6 mos post-surgery, on TAM for at least 6 mos and plan to continue, or at least 6 mos post-chemo	125/96	1,500 mg/d decreased testosterone by 23% (p < 0.01)
**(B) Primary prevention trials** (210)				
Breast Phase I ACTRN12610000219088	500 mg/d for 1 week followed by 1,000 mg/d for 4 weeks prior to reduction mammoplasty	Women age 40–60	60	AMPK signaling and aromatase expression
Breast Phase II NCT02028221	850 mg for 1 month followed by 850 mg bid for an additional 11 months vs. placebo	Premenopausal women age 30–45 with BMI of 25 or greater and have metabolic syndrome	150	Change in breast density from baseline at 6 and 12 months
Breast Phase II NCT01793948	850 mg qd for 30 days and bid for 11 months vs. placebo	Post-menopausal and high risk for breast cancer with BMI ≥25 or and high breast mammographic density	24	Changes in phosphorylated proteins by RPPM
Breast Phase III NCT01905046	850 mg qd for 4 weeks followed by 850 mg bid vs. placebo for 24 months. Placebo group may cross over to metformin for months 13–24.	Premenopausal, BMI ≥25, prior AH, LCIS, or DCIS, >1.66% Gail or known BRCA carrier, and cytological atypia	125/96	Endpoint: Regression of atypia at 12 and 24 mos Endpoint: Changes in phosphorylated proteins by RPPM

*IBC, invasive breast cancer; DCIS, ductal carcinoma in situ; qd, one a day; bid, twice a day; tid, three times a day; Tam, Tamoxifen; BMI, body mass index; HOMA, Homeostasis Model Assessment; CNB, core needle biopsy*.

*AH, atypical hyperplasia; LCIS, lobular carcinoma in situ; DCIS, ductal carcinoma in situ; qd, one a day; bid, twice a day; tid, three times a day; Tam, Tamoxifen; BMI, body mass index; RPPM, reverse phase proteomic microarray profiling*.

### Pre-surgical Assessment

Hyperglycemia and insulin resistance are linked to increased perioperative morbidity in patients with and without diabetes mellitus. Surgery itself induces transient insulin resistance, which worsens with the extent and duration of the procedure, leads to hyperglycemia, and contributes to post-operative complications (221, 222). In comparison to diabetic patients with post-operative hyperglycemia, non-diabetic patients with post-operative hyperglycemia have worse post-operative outcomes including death (223, 224), which may relate to undiagnosed, uncontrolled diabetes mellitus and/or heightened stress response to surgery.

Based on analyses of the American College of Surgeons National Surgical Quality Improvement Program (NSQIP) database 2010–2015, diabetic patients undergoing partial, total, or subcutaneous mastectomy were at greater risk of early post-operative surgical site infection—both superficial (partial and total mastectomy) and deep (total and subcutaneous) tissue infections (225). For diabetic women undergoing breast reconstruction, NSQIP data showed increased superficial surgical site infection with delayed but not immediate implant based procedures (225) and increased deep incisional infection, wound dehiscence, and post-operative length of stay with free flap reconstruction (226). In a retrospective study of diabetes and impact on complications in breast cancer patients undergoing mastectomy with immediate reconstruction, women with diabetes had significantly increased incidence of delayed wound healing with implant-based reconstruction but not autologous procedures (227).

Given the high prevalence of undiagnosed pre-diabetes and diabetes in the United States and worldwide, preoperative screening to identify such patients prior to surgical intervention is warranted. Numerous studies have investigated the utility of preoperative testing via HgbA1c in addition to the usual practice of random or fasting blood glucose levels. An observational cohort study of inpatient gastrointestinal surgical procedures at Veterans Affairs hospitals from 2007 to 2014 suggested that knowledge of elevated preoperative HgbA1c led to greater perioperative vigilance in monitoring and treating hyperglycemia and improved clinical outcome (228). Using HgbA1c to categorize surgical patients as diabetic, prediabetic, or normoglycemic, Yong et al. demonstrated that diabetic patients (based on prior diagnosis or HgbA1c ≥ 6.5%) had increased post-operative complications including higher risk of mortality at 6 months; prediabetes (HgbA1c 5.7–6.4%) was not associated with increased risk for adverse surgical outcomes (229). In a prospective database study of emergency general surgery cases, patients with HgbA1c measurements ≥6% within the prior 3 months had increased risk for development of in-hospital complications (major 12% vs. 4%, minor 24% vs. 7%) irrespective pre-operative serum glucose or preexisting diagnosis of diabetes (230). The risk for developing major complications after emergency surgery was four times higher for patients presenting with both HgbA1c ≥ 6% and glucose ≥200 (230). Routine HgbA1c level screening has been adopted for high risk surgeries that involve a patient population at high risk for pre-diabetes/undiagnosed diabetes, such as joint arthroplasty (231).

In a review of 20 studies regarding perioperative glycemic control in diabetic patients and post-operative complications, elevated preoperative HgbA1c was not associated with increased post-operative morbidity and mortality in 20 selected studies; the authors, however, noted the need for good quality evidence in view of the heterogeneous, retrospective studies, and small patient numbers (232). To evaluate 2001–2013 evidence that preoperative testing of blood glucose and HgbA1c might affect outcome measures in elective non-cardiac surgery, a systematic review concluded that preoperative blood glucose and HgbA1c tests are only indicated in non-diabetic patients upon clinical situation of an abnormality or elevated risk such as vascular and orthopedic surgery (233). A systematic review of observational studies of non-diabetic patients with elevated preoperative HgbA1c showed increased post-operative complications in non-diabetic patients in four of the six reports, lending support for use of suboptimal HgbA1c levels as a modifiable marker of adverse post-operative outcomes (234). As noted by the authors of these systematic reviews, limitations relate to the paucity of high-quality studies, lack of randomized controlled trials, and high heterogeneity of available studies.

### Factors That Promote Insulin-Resistance During Breast Cancer Chemotherapy

Women receiving breast cancer chemotherapy are extensively monitored for electrolyte and liver function abnormalities and neutropenia. While serum glucose is routinely tested, there is minimal attention to development of insulin-resistance during breast cancer treatment. This is a missed opportunity and represents an important opportunity for improving cancer care. Given the key role that insulin signaling plays in cancer processes, it is logical to ensure that women who are treated for breast cancer do not develop insulin-resistance.

Women who are treated for breast cancer experience stress, abnormal sleep patterns, overeating, administration of steroids, loss of body image, gastrointestinal disturbances, and immune suppression (235). Breast cancer survivors experience many treatment-associated changes, including weight gain, reduced physical activity levels, and metabolic syndrome (235–238). Metabolic syndrome is diagnosed when a woman has any three of the five following components: (1) waist circumference ≥80 cm (32 inches); (2) elevated triglycerides ≥150 mg/dL or on drug treatment for elevated hypertiglyceridemia; (3) reduced high-density lipoprotein cholesterol <40 mg/dL; (4) elevated blood pressure ≥130/85 mm Hg or on antihypertensive drug treatment; (5) elevated fasting blood glucose ≥100 mg/dL or on drug treatment for elevated glucose) (236–238). Metabolic syndrome and its associated factors, including obesity, physical inactivity, hyperinsulinemia, insulin resistance, elevated inflammatory biomarkers, and altered adipokines, are all linked with increased risks of breast cancer, all-cause mortality, and increased risk of breast cancer recurrence (236–238).

Joanne Mortimer's team from City of Hope prospectively investigated in pre- and post-menopausal women with Stage I–III breast cancer, the impact of neo-adjuvant chemotherapy on development of insulin-resistance and metabolic syndrome (235). A total of 86 previously healthy women (46 premenopausal; 40 post-menopausal) were tested for the components of metabolic syndrome before and after completion of neo-adjuvant chemotherapy. Also measured were HgbA1c, insulin-resistance, and C-reactive protein. The study demonstrated that all individual components of metabolic syndrome were statistically increased after chemotherapy (*p* < 0.01) (235). Body weight, percent body fat, fat mass, C-reactive protein, and HgbA1c were all increased as well (*p* < 0.01) (235). Taken together, this study highlights an important missed opportunity for optimizing cancer care and prevention of breast cancer recurrence.

## Conclusions

While diabetes/insulin-resistance and breast cancer are distinct diseases, insulin-signaling plays a central role in both illnesses. Insulin activates key cancer processes including EMT, tissue inflammation, motility, and angiogenesis. There are key opportunities to impact and prevent hyperinsulinemia during breast cancer prevention, surgical assessment, and chemotherapy. While it is not standard of care to test for insulin-resistance during the course of breast cancer screening and treatment, it is standard of care to screen and test high risk women for insulin-resistance as part of whole woman care. Given the important role insulin signaling plays in driving signaling pathways that promote aggressive cancer biology, more attention should be paid by cancer physicians to screening and treating insulin resistance.

## Author Contributions

VS organized the manuscript and wrote many of the sections. JM wrote the section on breast cancer medical oncology treatment and insulin resistance. LY wrote the sections on Asians and surgical complications and insulin resistance. RN cowrote the section on type 2 diabetes, progressive beta-cell failure, and decline in insulin production. ED cowrote the section on type 2 diabetes and progressive beta-cell failure.

### Conflict of Interest

The authors declare that the research was conducted in the absence of any commercial or financial relationships that could be construed as a potential conflict of interest.
